# Can public education campaigns equitably counter the use of substandard and falsified medical products in African countries?

**DOI:** 10.1093/heapol/czaf004

**Published:** 2025-01-18

**Authors:** Janelle M Wagnild, Samuel Asiedu Owusu, Simon Mariwah, Victor I Kolo, Ahmed Vandi, Didacus Bambaiha Namanya, Rutendo Kuwana, Babatunde Jayeola, Vigil Prah-Ashun, Moji Christianah Adeyeye, James Komeh, David Nahamya, Kate Hampshire

**Affiliations:** Department of Anthropology, Durham University, South Road, Durham DH1 3LE, UK; Directorate of Research, Innovation and Consultancy, University of Cape Coast, New Administration Block, Cape Coast, Ghana; Department of Geography and Regional Planning, University of Cape Coast, Faculty of Social Sciences, Cape Coast, Ghana; School of Public Health, University of Medical Sciences, Ondo City, Ondo State PMB 536, Nigeria; Department of Community Health and Clinical Sciences, Njala University, Bo Campus, Bo, Sierra Leone; Ministry of Health, PO Box 7272, Lourdel Road, Kampala, Uganda; Incidents and Substandard/Falsified Medical Products Team, World Health Organization, Avenue Appia 20, Geneva 1211, Switzerland; World Health Organization, Cité du Djoué, P.O.Box 06, Brazzaville, Republic of Congo; Food and Drugs Authority, 17 Nelson Mandela Ave, Accra, Ghana; National Agency for Food and Drug Administration and Control, Plot 2032, Olusegun, Obasanjo Way, Zone 7, Wuse, Abuja, Nigeria; Pharmacy Board of Sierra Leone, New England Ville, Freetown, Sierra Leone; National Drug Authority, PO Box 23096, Kampala, Uganda; Department of Anthropology, Durham University, South Road, Durham DH1 3LE, UK

**Keywords:** Ghana, Nigeria, Sierra Leone, Uganda, medicine quality, risk communication

## Abstract

Substandard and falsified (SF) medical products are a serious health and economic concern that disproportionately impact low- and middle-income countries and marginalized groups. Public education campaigns are demand-side interventions that may reduce the risk of SF exposure, but the effectiveness of such campaigns, and their likelihood of benefitting everybody, is unclear. Nationwide pilot risk communication campaigns, involving multiple media, were deployed in Ghana, Nigeria, Sierra Leone, and Uganda in 2020–21. Focus group discussions (*n* = 73 with *n* = 611 total participants) and key informant interviews (*n* = 80 individual interviews and *n* = 4 group interviews with *n* = 111 total informants) were conducted within each of the four countries to ascertain the reach and effectiveness of the campaign. Small proportions of focus group discussants (8.0–13.9%) and key informants (12.5–31.4%) had previously encountered the campaign materials. Understandability varied: the use of English and select local languages, combined with high rates of illiteracy, meant that some were not able to understand the campaign. The capacity for people to act on the messages was extremely limited: inaccessibility, unavailability, and unaffordability of quality-assured medicines from official sources, as well as illiteracy, constrained what people could realistically do in response to the campaign. Importantly, reach, understandability, and capacity to respond were especially limited among marginalized groups, who are already at the greatest risk of exposure to SF products. These findings suggest that there may be potential for public education campaigns to help combat the issue of SF medicines through prevention, but that the impact of public education is likely to be limited and may even inadvertently widen health inequities. This indicates that public education campaigns are not a single solution; they can only be properly effective if accompanied by health system strengthening and supply-side interventions that aim to increase the effectiveness of regulation.

Key messages

- Evaluation of a pilot risk communication campaign about substandard and falsified medicines in Ghana, Nigeria, Sierra Leone, and Uganda showed that its reach and understandability were limited and many were unable to act on the campaign’s advice due to structural barriers.
- These challenges were inequitably distributed, with marginalized groups more likely to be unable to receive, understand, and respond to the campaign, highlighting the potential for public health communications like this to widen health inequities.

## Introduction

### The inequitable burden of substandard and falsified medical products

Substandard and falsified (SF) medical products are a serious health and economic concern, particularly in low- and middle-income countries (LMICs). ‘Substandard’ medical products have been authorized by national authorities but fail to meet either quality standards or specifications, or both; ‘falsified’ medical products deliberately misrepresent a drug’s identity, composition, or source (World Health Assembly 2017). The World Health Organization estimates that over 10% of medicines in LMICs are substandard or falsified (WHO 2017).

Potential exposure to SF medical products is unevenly distributed between and within countries. At the global level, SF medicines have been discovered and reported in all regions of the world (WHO 2017), but LMICs—and sub-Saharan Africa in particular—are disproportionately impacted by SF medical products (Ozawa et al. 2018). Within countries, marginalized groups, e.g. those living in rural areas and those of lower socioeconomic status, are at particular risk of SF medical products and their effects (Evans et al. 2019, Wagnild et al. 2024). These differences represent ‘health inequities’, defined as unfair and avoidable differences in health-related outcomes and experiences based on socially stratified circumstances, such as place of residence, gender, socioeconomic status, and education (O’Neill et al. 2014).

### Ensuring medicine quality through equitable supply- and demand-side interventions

Both supply- and demand-side interventions are necessary to address the issue of SF medical products. Supply-side measures at the international and national levels, such as increasing the security of supply chains and strengthening regulatory capacity, aim to prevent SF medical products from reaching consumers (Hamilton et al. 2016, WHO 2017). On the demand side, education and awareness about SF products is viewed as a first step in preventing potential exposure to SF medical products (WHO 2017). This includes risk communication campaigns, which aim to raise public awareness about the existence of SF medicines and steps that can be taken to avoid SF medical products. These steps typically involve seeking professional advice before taking medicines, avoiding unauthorized medicine vendors, and visually inspecting the medicine and packaging before use (WHO 2018).

**Figure 1.**
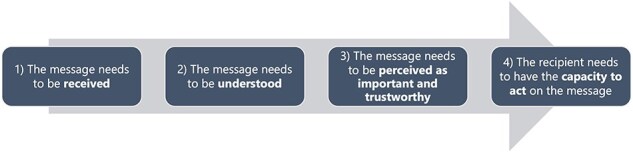
Analytical framework.

Whether SF risk communication campaigns can effectively impact public practices in this way is unknown. Campaigns must reach their intended audience, but simply receiving a message does not necessarily translate into modified practices (Dusetzina et al. 2012, Cohn 2014, Kelly and Barker 2016). Messages must also be understood by the target audience, be perceived as important and trustworthy, and crucially, the recipient must have the capacity to act on the messages (Fig. 1). The latter step is especially pertinent in the context of medicine use in LMICs: structural barriers (‘Structural barriers’ refer to constraints experienced due to social factors and arrangements over which the individual has limited or no control; these can include, e.g. gender, geographical location, and access to education) to accessing quality-assured medicines can limit what people can realistically do in response to an information campaign (Wagnild et al. 2024).

In addition, there is a clear possibility that risk communication campaigns can inadvertently widen health inequities. Previous work has shown that structurally disadvantaged groups (including women, those living in rural areas, those with low education, and those not in paid work) are less likely to be aware of SF medical products and are also more likely to experience difficulties accessing quality-assured medical products (Wagnild et al. 2024). Communication inequalities, whereby marginalized groups are less likely to receive, understand, and be able to act upon public health communications (Viswanath et al. 2007, Häfliger et al. 2023), can exacerbate and reinforce this inequity. Thus, when evaluating whether SF risk communication campaigns can be effective, it is critical to also consider for whom campaigns might be effective, and what implications this may have for widening health inequities.

### Is SF medicine public education (equitably) effective? The current study

To understand the extent to which a risk communication campaign on SF medical products can be effective and equitable, this paper evaluates the reach and effectiveness of a pilot SF risk communication campaign conducted in four African countries (Ghana, Nigeria, Sierra Leone, and Uganda). This evaluation is guided by the framework positing that, in order for a risk communication to be effective, the following four criteria must be met: it must (i) reach the target audience, (ii) be understood by them, (iii) be perceived as important and trustworthy, and (iv) the target audience must have the capacity to act on the messages.

This evaluation therefore aims to address the following questions:

1. Did the campaign reach the intended audience?
2. Was the campaign understood by the intended audience?
3. Were the campaign messages perceived as important and trustworthy by the intended audience?
4. Did the target audience have the capacity to act on the campaign messages?
5. Were there clear inequities in any of the above steps (i.e. was the campaign more likely to reach and/or benefit advantaged groups)?

## Background and contextual information

### The four countries

Key social and economic indicators for each of the countries are shown in Table 1. According to the World Bank, Ghana and Nigeria are classified as lower-middle income economies, while Sierra Leone and Uganda are considered low-income economies.

**Table 1.** Key social and economic indicators for the four countries

Indicators	Ghana	Nigeria	Sierra Leone	Uganda
Population	32 833 031	218 541 212	8 605 718	47 249 585
Rural population (% of total population)	42	46	66	74
GDP per capita (USD)	2363.3	2184.4	461.4	964.2
Under-5 mortality (per 1000 live births)	44	111*	105*	42*
Life expectancy at birth (years)	64	53^a^	60^a^	63
Out-of-pocket expenditure (% of all health expenditure)	30.8^b^	74.7^b^	55.7^b^	37.5^b^
Physicians (per 1000 people)	0.2^b^	0.4^c^	0.1^c^	0.2^b^
Estimated proportion of the population covered by health insurance^d^ (%)	58.2	2.3	1.5	1.5
Literacy rate (% of people aged ≥15 years)	Overall: 80^b^ Males: 84^b^ Females: 76^b^	Overall: 62^c^ Males: 71^b^ Females: 53^c^	Overall: 49 Males: 56 Females: 41	Overall: 81 Males: 85 Females: 77
Access to electricity (% of population)	85.1	60.5	29.4	47.1

Data from World Bank (2022 unless otherwise indicated).

a Data from 2021.

b Data from 2020.

c Data from 2018.

d Data from Barasa E, Kazungu J, Nguhiu P, Ravishankar N. Examining the level and inequality in health insurance coverage in 36 sub-Saharan African countries. *BMJ Glob Health* 2021 Apr;6(4):e004712.

In all four countries, medicines are legally available both in public-sector health facilities and private-sector retail outlets. In Ghana, Nigeria, and Sierra Leone, patients using public-sector health facilities must pay user fees for services, including consultations, laboratory tests, and medicines. Some users are exempt from paying these fees, including those enrolled in the National Health Insurance Schemes (in Ghana and Nigeria) and certain groups, e.g. children <5 years and pregnant women (in Nigeria and Sierra Leone). In Uganda, primary care services in the public sector, including consultations and medicines, are free of charge. In all four countries, medicines are legally available in two different kinds of licensed private-sector retail outlets: (i) pharmacies, which sell prescription-only, pharmacy, and over-the-counter medicines; and (ii) licensed over-the-counter medicine shops, commonly referred to as ‘drug stores’, ‘chemists’, ‘chemical shops’, or ‘clinics’ depending on the country, which are permitted to sell over-the-counter medicines only. In all four countries, we observed during fieldwork that medicines are also available illegally in informal markets.

### The pilot campaigns

Pilot risk communication campaigns led by the World Health Organization and local National Medical Regulatory Authorities (NMRAs) were deployed nationwide in Ghana, Nigeria, Sierra Leone, and Uganda in 2020–21. In all four countries, public-facing posters were produced (see Supplementary File Figure S1) and displayed at various sites across the countries, alongside a set of posters aimed specifically at healthcare professionals. All four campaigns also included a radio component: usually a short jingle, which aired over a period of 1–3 months. TV adverts were also deployed in all countries except Ghana, and bumper stickers were used in Sierra Leone. All materials were produced in English and some of the major local languages spoken in each country (see Supplementary File Table S1).

There was some variation in the specific contents of each campaign component within and between countries (see Supplementary File Figure S1), but the materials broadly advised the public to take the following steps:

- Speak with a healthcare professional when feeling unwell (applies to all countries except Uganda).
- Only buy medicines from licensed pharmacies or over-the-counter medicine sellers (in all countries except Uganda).
- Check medicine packaging for poor condition, spelling mistakes, and unclear labels (all countries).
- Check the medicine packaging for the batch number, manufacturing date, expiry date, manufacturer’s address, and NAFDAC number (all countries, except the NAFDAC number which was only in Nigeria).
- Ensure the medicine does not look unusual or broken (Sierra Leone and Uganda only).
- Report any suspicious medicines, adverse reactions to a medicine, or unlicensed sellers to the local NMRA using the phone and/or WhatsApp number provided on the poster (all countries).

## Methods

### Study design

The study design was qualitative, comprising three components. Focus group discussions (FGDs) were conducted with members of the public across multiple sites per country, to ascertain the reach of the campaign materials and to gather views on the campaign and relevant medicine-related experiences. The use of FGDs allowed us to obtain a varied and contrasting range of views/experiences on these topics (Konopinski 2014). Face-to-face key informant interviews (KIIs) were conducted at each site, with individuals whose position in the community afforded them unique access to the topic (Pahwa et al. 2023), allowing us to establish broad parameters of medicine purchase, use, and decision-making in each location. This was complemented by ethnographic observation throughout, particularly in informal markets where medicines were being sold. Methodological rigour was ensured by: (i) triangulating these different information sources; (ii) using thick description (providing contextual information and direct quotations, to allow readers to assess our interpretations), and (iii) involving local researchers in data collection and analysis, to ensure cultural/contextual relevance.

### Study settings

Fieldwork took place across 18 sites in total (9 urban, 9 rural), in contrasting regions within each country between November 2022 and October 2023. Purposive stratified selection of study sites was used to ensure relevant variation in the sample (see Robinson 2013). This followed a three-stage process within each country: first, the selection of geographically- and culturally distinctive regions, followed by the selection of administrations/districts/states, and then urban and rural sites (see Supplementary File Table S2) as there are well-established urban/rural differences in medicine-related knowledge and access (Wagnild et al. 2024).

Access to each study site was gained through a ‘gatekeeper’ such as a community leader or local healthcare manager. The purposes and procedures of the study were explained to the gatekeepers in-person ahead of the fieldwork and initial consent for study activities to take place within the community was granted. The gatekeepers helped recruit study participants, and some also agreed to take part in an interview.

### Data collection

#### Focus group discussions (FGDs)

A total of 73 FGDs were conducted, comprising 611 individuals: 16 groups in Ghana (totalling *n* = 137 participants), 23 groups in Nigeria (*n* = 174 participants), 16 groups in Sierra Leone (*n* = 115 participants), and 18 groups in Uganda (*n* = 185 participants). FGD participants were recruited with the help of a gatekeeper to ensure that a diverse range of views was represented; groups were then stratified by gender and/or age as appropriate within each country so participants could speak freely. FGDs were conducted in English, through translation, or in a mixture of English and local languages to accommodate participants’ preferences.

FGDs were structured by first discussing experiences of medicines within the community, including where people obtained medicines, why, and any concerns they had (see Supplementary File Table S3). We then showed the campaign materials to the group and asked participants one by one whether they had ever seen the poster, heard the jingle, or seen the TV advert (as appropriate in each country), and these responses were tabulated. This was followed by discussion of the materials, participants’ responses and reactions to them, their thoughts about how effective they were likely to be in their community, and what adjustments or strategies might make it more effective going forward. On average, each FGD lasted ∼45 min to an hour.

#### Key informant interviews

A total of 111 key informants were interviewed. Eighty individual KIIs were conducted with community leaders and local healthcare professionals (including assemblymen, traditional leaders, religious leaders, hospital managers and in-charges, nurses, midwives, village health team members, pharmacists, medicine sellers, and district social mobilization officer). Additionally, four group interviews with key informants (with *n* = 5 medicine retailers and *n* = 5 healthcare workers in Nigeria; with *n* = 13 healthcare professionals, *n* = 8 community stakeholders in Sierra Leone). The majority of KIIs were conducted in English but, where the respondent preferred they were conducted in local languages and subsequently translated into English for analysis.

The structure of these interviews was broadly similar to that of the FGDs, where views about medicine use in the community were gathered first before showing and discussing the campaign materials (see Supplementary File Table S4), but the exact wording and order of questions were adapted according to each individual’s position (e.g. religious leader compared with health-worker).

#### Ethnographic observation

The FGDs and KIIs were supplemented by ethnographic observation throughout the fieldwork, including the observation of campaign materials in study sites and people’s reactions to them, as well as visit to informal markets to observe any pharmaceuticals being sold. The findings of these observations will be reported in-depth in a separate publication.

### Data preparation and analysis

The FGDs and KIIs were transcribed from audio recordings and translated into English where necessary. The transcripts were uploaded to NVivo software, where thematic analysis was undertaken (Clarke and Braun 2016). The first stage of analysis entailed broad deductive coding of qualitative data, based on the framework described in the ‘Introduction’ section: the target population needs to have received the message, understood it, perceived it as important and trustworthy, and had the capacity to act meaningfully on it. In the second stage, the data were further coded inductively into themes and sub-themes based on the patterns that emerged in the data. Initial coding was conducted independently by two of the authors (J.W. and K.H.), with discussion at regular intervals to ensure consistency and reliability. Data on the reach of the campaign materials were ascertained by tabulating responses within each FGD and interview.

Findings were shared with key stakeholders through presentations during the analysis and through circulation of manuscript drafts, for the purposes of dissemination and cross-checking factual information.

### Ethical considerations

The study received ethical approval from the Research Ethics Committee in the Department of Anthropology at Durham University and from ethics committees within each of the four countries. As noted earlier, initial group consent was sought from the community gatekeepers, in line with the usual protocols for working in these locations. Fully informed individual consent was then sought verbally from each research participant before research activities began. No personally identifiable data were collected during this project.

## Results

### Description of study sample

A description of the FGD participants can be found in Table 2; a detailed list of key informants can be found in the Supplementary File (Table S5). In the FGD sample, there were more women than men (54% vs 46%) and slightly more participants in rural locations than in urban (52% vs 48%). The mean age of FGD participants was 35.4 years (standard deviation 12.4 years; range 16–82 years). The majority of key informants were healthcare professionals (64%, vs 36% community leaders) and around two-thirds (66%) were men.

**Table 2.** Description of focus group discussants

	Men	Women	Total
**Ghana**			**137 (22.4)**
Greater Accra (*n *= 56)			
Urban	15 (2.5)	14 (2.3)	29 (4.7)
Rural	7 (1.1)	20 (3.3)	27 (4.4)
Upper East (*n *= 81)			
Urban	13 (2.1)	23 (3.8)	36 (5.9)
Rural	25 (4.1)	20 (3.3)	45 (7.4)
**Nigeria**			**174 (28.5)**
Federal Capital Territory (*n* = 112)			
Urban	22 (3.6)	26 (4.3)	48 (7.9)
Rural	30 (4.9)	34 (5.6)	64 (10.5)
Kwara State (*n* = 62)			
Urban	20 (3.3)	28 (4.6)	48 (7.9)
Rural	7 (1.1)	7 (1.1)	14 (2.3)
Sierra Leone*			**115 (18.8)**
Western Area Rural District (*n *= 48)			
Urban	15 (2.5)	12 (2.0)	27 (4.4)
Rural	6 (1.0)	15 (2.5)	21 (3.4)
Port Loko District (*n *= 49)			
Urban	8 (1.3)	8 (1.3)	16 (2.6)
Rural	16 (2.6)	17 (2.8)	33 (5.4)
Bo District (*n *= 18)			
Rural	6 (1.0)	12 (2.0)	18 (2.9)
**Uganda**			**185 (30.3)**
Mbale (*n* = 104)			
Urban	26 (4.3)	25 (4.1)	51 (8.3)
Rural	28 (4.6)	25 (4.1)	53 (8.7)
Mbarara (*n *= 81)			
Urban	18 (2.9)	21 (3.4)	39 (6.4)
Rural	19 (3.1)	23 (3.8)	42 (6.9)
**Total**	**281 (46.0)**	**330 (54.0)**	**611 (100)**

Data shown as *n* (%) of total number of FGD participants.

### Reach of the campaigns

Of the 611 FGD participants, only 66 (10.8%) reported having previously encountered any of the campaign materials (four of the 66 who had previously encountered the campaign had encountered more than one medium; two respondents in Nigeria reportedly saw the TV advert and had heard the radio jingle, and two in Uganda had seen both the poster and the TV advert). The proportions of FGD participants within each country who had previously encountered any campaign materials were broadly similar between countries: 11 (8.0%) participants in Ghana, 17 (9.8%) in Nigeria, 16 (13.9%) in Sierra Leone, and 22 (11.9%) in Uganda.

**Figure 2.**
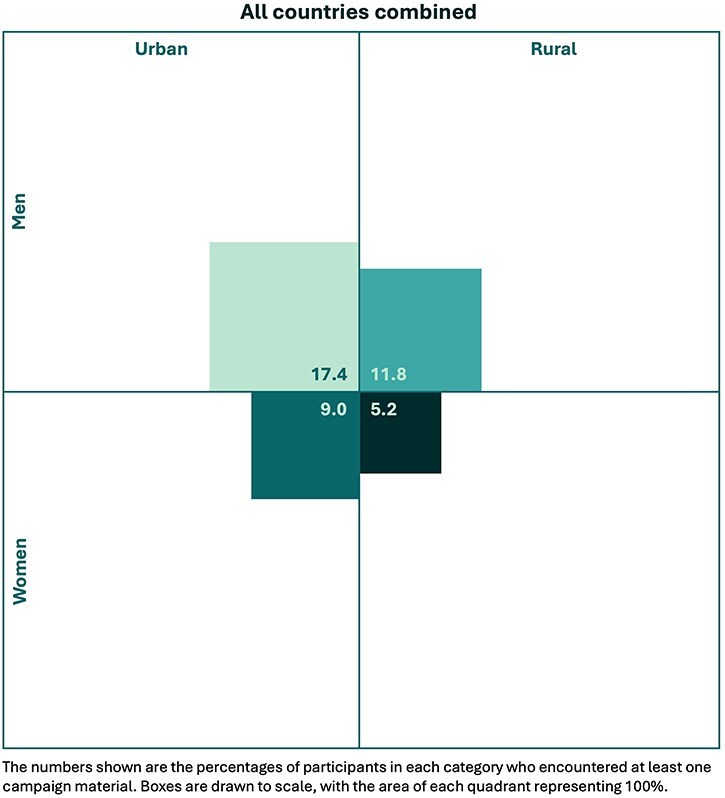
Distribution of campaign recipients according to gender and urban/rural locality.

Different media were more likely to have been viewed in different countries; e.g. the poster was the most viewed material in Sierra Leone (seen by eight FGD participants) but the least viewed in Nigeria (only one participant; see Supplementary File Table S6). Overall, men living in urban areas were the most likely to have encountered at least one of the campaign materials (17.4%) while women living in rural areas were the least likely to have encountered any of the campaign materials (5.2%; Fig. 2).

Of the 111 key informants interviewed, 25 (22.5%) reported having previously encountered any of the campaign materials (see Supplementary File Table S7). (Ten of the 25 who had previously encountered the campaign had encountered more than one medium; five in Nigeria and two in Sierra Leone had heard the radio jingle and seen the TV advert; two in Ghana and one in Sierra Leone had seen the poster and heard the radio jingle). This ranged from 12.5% (*n* = 2) in Uganda to 31.4% (*n* = 11) in Nigeria, with Sierra Leone (17.8%, *n* = 7) and Ghana (23.8%, *n* = 5) in between. Over a third of key informants in Nigeria had previously heard the radio jingle and seen the TV advert. No key informants in Uganda had seen the poster or heard the radio talk show, although 20% indicated they had seen the TV advert before.

### Understandability of the messages

Participants in all four countries raised concerns about the understandability of the messages in the context of their communities. The posters were produced in English and the literacy rates (in English) were variable: women, older people, and those in rural areas were less likely to be able to read the poster, compared with men, younger people, and urban-dwellers. The requirement of (English language) literacy to understand the poster was highlighted repeatedly as a barrier to understanding in all four countries:

This information on the posters, for instance, they are very abstract for someone like me [who cannot read]. Most of the people in this community are also illiterate so they are not able to read. (24-year-old female, rural Bo, Sierra Leone)

To help circumvent the need for literacy, the posters in all four countries also included photographs in addition to text (see Supplementary File Figure S1), depicting damaged medicines or medicine packaging with a red ‘X’ over it. The posters in Nigeria and Sierra Leone additionally included photos of intact medicine packaging with a green checkmark. Participants noted that, although many understood that the photographs on the poster were conveying ‘bad’ and ‘good’ medicines through the red ‘Xs’ and green checkmarks, respectively, those who could not read struggled to understand from the picture exactly what made the ‘bad’ medicine problematic:

There are some who cannot read English, and then there are people who cannot read at all. So this picture tells a lot but there is a way to improve… An illiterate person does not know what is marked and cancelled. (Female, urban Federal Capital Territory, Nigeria)

Language barriers also inhibited understandability. Radio jingles/talk shows and TV adverts were produced in English and in at least one local language. Although English is the official language in three of the countries, it is not understood by everyone, particularly those who are older, have not received formal education, and live in rural areas:

When I would see these things on radio [sic], since they are speaking in English, I would think it is for educated people, not for illiterates like me. (46-year-old male, rural Mbale, Uganda)

The translation of the radio and TV adverts into one or more major local languages enabled some to understand the messages, but those who did not speak that particular local language were unable to understand. For example, the radio jingle was only translated into Twi in Ghana; while Twi is the most widely spoken local language in Ghana, it is more widely spoken in the southern and central regions of the country. Participants in the north of the country, where other local languages predominate, struggled to understand the jingle:

The main problem with the jingle you played to us is that it is not in our local language so we don’t get the message. It shouldn’t just be in the Twi language. (34-year-old male, rural Upper East, Ghana)

### Perceived importance of the messages

Participants generally indicated they felt that the topic of SF medicines and the communication campaign were very important. There was broad awareness of SF medical products among participants, who regarded SF products as ‘a big problem in this community’ and ‘a cause for concern’. The sources of this knowledge varied between countries. In Nigeria, participants indicated that regular advertisements from NAFDAC and routine education provided by health facilities made them aware of SF medicines. In Sierra Leone, awareness seemed to predominantly stem from personal encounters with presumed SF products. In almost half of the FGDs, at least one participant described an experience (either directly or a family member) with a suspected SF product, with effects ranging from ‘feeling differently’ and then realizing that the consumed medicine was expired, to more serious outcomes including the requirement of immediate medical intervention and even one reported death:

My uncle once bought medicine from one of these peddlers. He immediately started exhibiting some strange symptoms after taking the medicine; he was only saved by an intervention from one of the health professionals at the health centre. (22-year-old male, rural Port Loko, Sierra Leone)

### Capacity to act on the messages

The actions encouraged in this campaign included speaking to healthcare professionals when feeling ill, obtaining medicines from licensed pharmaceutical outlets, and checking features of the medicine and its packaging (including expiry dates and spelling mistakes) to ensure the medicine was not obviously fake or damaged. Participants identified a range of constraints that could make it difficult for people (including themselves) to act on the campaign’s messages, even when they recognized the messages to be important. These constraints fell into the broad categories of (i) difficulties accessing and utilizing health facilities, (ii) difficulties accessing and utilizing licensed retail outlets, and (iii) difficulties checking medicines.

#### Difficulties accessing and utilizing health facilities

Overwhelmingly, the most frequently mentioned barrier to following the campaign advice to speak with a healthcare professional when feeling unwell in Ghana, Nigeria, and Sierra Leone was poverty [Poverty was also a barrier experienced by participants in Uganda but, as healthcare is free in Uganda, this pertained to accessing medicines in the private sector (detailed in the following section)]. For those without insurance or an exemption (the vast majority in all countries except Ghana; Table 1), the total cost for a visit to a government facility could be prohibitive. For example, participants in Sierra Leone said that, in their experience of attending the hospital, they had to pay between SLL150 000 and SLL300 000 (∼USD7 and USD18 at the time of fieldwork), while participants in Nigeria noted that costs of attending the government facility could add up to NGN1800 to NGN5000 (∼USD2.25 to USD6.25 at the time of fieldwork). These costs were simply unaffordable for most:

Most of these facilities like the hospital, when you are sick, when you go there, it is very, very expensive … If I’m sick I cannot go to the hospital to try at least to know first what is the problem. I’ll try to treat my symptoms before going to the hospital … As a result of poverty, I will not go. (30-year-old male, urban Port Loko, Sierra Leone)

Therefore, in Ghana, Nigeria, and Sierra Leone, many respondents said that instead of first speaking with a healthcare professional at the health centre, they preferred to go straight to the drug store to get medicines. This was viewed as a much cheaper option, primarily because going to the drug store only incurred the cost of the medicine itself without the additional (and sometimes unpredictable) fees for consultations and tests in the government facilities. Several participants in Ghana and Nigeria poignantly stated that they knew what they ‘should’ do, in terms of attending health facilities when they are ill. However, in practice, many were unable to do this because they lacked the resources:

It is not that we are very happy with going to the drugstore, but because we don’t have money to go to the hospital, we have to settle on the drugstore. Sometimes the drug store will not completely treat the illness, but we don’t have a choice. (43-year-old male, urban Greater Accra, Ghana)

Many of them are intending to [go to the health centre first], but lack of money does not allow them to seek professional healthcare advice. (Key informant, rural Kwara, Nigeria)

Another major barrier to acting on the campaign’s advice was the inaccessibility of health facilities. In Ghana, Sierra Leone, and Uganda, the nearest health centre or hospital could be very far away, particularly for those in rural communities. Some participants had no health facilities in their communities, requiring them to travel substantial and prohibitively expensive distances to nearby towns:

We have no areas where we can get medicine. There is no specific place [to get medicines] in this town. We have to take our people to [the nearest] town over 16 miles [away]. (18-year-old male, rural Bo, Sierra Leone)

Unavailability of medicines at the health facilities was another common constraint widely experienced in Ghana, Sierra Leone, and Uganda. Participants in all study sites in these countries raised stock-outs as a major impediment to accessing medicines, describing the common circumstance of attending the health facility only to receive a prescription to go and buy the medicine elsewhere:

Sometimes we go to the government hospitals and we don’t find the medicine. They just prescribe for you and they tell you to go and buy. (Male, age unknown, urban Mbale, Uganda)

The frequency of stockouts of medicines in government facilities in Ghana, Sierra Leone, and Uganda understandably dissuaded many respondents from attending the health facility in the first place, knowing they would often have to wait in long queues only to ultimately be sent elsewhere to get the medicine they needed. Going straight to the drugstore was therefore often viewed as a more efficient use of time and resources:

You will go [to the hospital] to spend most of your time there only to be told by the health professionals that they do not have medicines at their pharmacy, so you should take the prescription and buy the medicines from the drug store. If that will be the case, why should we waste our time and other resources to go to the hospital when we can easily go to the drugstore for the same medicines without wasting any time there? (Male key informant, rural Upper East, Ghana)

#### Difficulties accessing medicines at licensed retail outlets

Participants often patronized licensed retail outlets, either after visiting a health facility (i.e. with a prescription due to stockouts) or as a direct route to circumvent the challenges described earlier. In some rural communities, licensed retail outlets could be a long distance away making them very challenging to access:

One of the difficulties is accessibility; where to get the medicines. In this community, we don’t have any drug stores. We have to go to [the next town] to buy medicines. (20-year-old male, rural Upper East, Ghana)

Unaffordability of medicines from licensed outlets was another key barrier. Financial constraints determined which medicine and how much medicine was purchased. Respondents spoke of the central role that money played in selecting which medicine to purchase from retailers:

There are times when we go to the drugstore and opt for the cheapest medicine. Assuming, you have a headache and the attendant gives you a range of medicines that can treat the headache, you choose the cheapest one because of financial challenges. (43-year-old male, urban Greater Accra, Ghana)

Many participants in Uganda, who attended the (free) health centre but were frequently given prescriptions to purchase medicines from the private sector due to stock-outs, described struggling to afford the medicine they were prescribed:

If I’m sick, I go to the health centre. After checking you, they tell you ‘go to buy drugs outside for this.’ I don’t have money. I come back home and I said ‘what can I do?’. But they are treating you, you have malaria, and you come back without even Panadol. They write for you to get this medicine to buy outside. We don’t have this. If you don’t have money, you come back and sit because treatment requires money … You cannot buy if the money is not there. (55-year-old female, urban Mbale, Uganda)

Financial constraints also contributed to a practice of underdosing, whereby too little active pharmaceutical ingredient (API) is consumed. It was widely reported that, when a customer was unable to afford a full dosage of medicine, medicine vendors would often dispense a partial dose, according to what could be afforded, rather than refusing to dispense altogether. As such, they were acting in the interests of alleviating customers’ suffering in the short term and/or keeping their businesses running (see Hampshire et al. 2022). For example, at a drug shop we visited in Uganda, a full course of antimalarial medicine (e.g. Coartem) cost UGX4000 (∼USD1). Those unable to afford this amount would often resort to purchasing an incomplete dose:

Money is also a problem because a person can come from the health centre. They need Coartem and Panadol which maybe is over UGX4000, when the person is coming with UGX1000. So what do you do? As a nurse…I explain to that person that the proper dosage is supposed to be UGX4000. You have brought 1000. What is the way forward now? That person will tell me, ‘Give me the first UGX1000 and then I will come back.’ When you give the first dose, that person does not come back. They feel a bit okay—So they don’t finish the dosage. And me, as a nurse, I know it is a problem of money. (Key informant, rural Mbale, Uganda)

Key informants in all four countries (especially Uganda) noted the risks of underdosing, both to the individual (diminished therapeutic value) and increasing the wider risk of antimicrobial resistance. Several interlocutors further noted that buying partial medicine doses because of poor affordability was likely to be a much more important driver of underdosing in their communities than the risk of medicines being substandard or falsified (containing too little API), as this FGD participant’s comment exemplify:

Underdose itself is a problem. You may go to the referral hospital, you may not find some drug there, so they refer you to appropriate pharmacies or clinics. The problem now we have said is poverty leading to that self-medication which has brought resistance. Now what could be the solution? The problem we are having here is poverty. That poverty leading to self-medication and underdosing and becoming resistant. How are we going to manage that poverty? So the problem is poverty, not fake drugs or whatever. (54-year-old male, rural Mbarara, Uganda)

#### Difficulties checking medicines

The third and final set of limitations to acting on the messages in the campaigns was the low literacy rate in some of the study communities. As noted earlier, two of the campaign’s messages were to check medicine packaging for spelling mistakes, expiry dates, and other written pieces of information; an additional campaign message was to obtain medicines only from licensed medicine sellers. Just as illiteracy was a barrier to understanding the campaign poster, it was also a barrier to following these pieces of advice. Lack of literacy made it very challenging to check aspects of the medicine that involved English words (e.g. spelling mistakes) or numbers (e.g. expiry dates):

If you can’t read and write, how can you check the dates and other information written on the medicine? So illiteracy is one thing that may prevent someone from obeying the message. (25-year-old female, rural Upper East, Ghana)

On the issue of expiry date, there are certain people that, when they are given the medicine, maybe because of the illiteracy, it is very hard for them to check the expiry date or manufacturing date and the batch number. It is hard for them. (25-year-old male, rural Mbale, Uganda)

However, regardless of literacy, several participants in Ghana and Sierra Leone commented on the physical aspects of the medicines that could help them determine whether the medicine was likely to be of good quality, if it was a medicine with which they were already quite familiar. For example, they knew what the appearance and texture of medicines they had previously taken (e.g. paracetamol) ought to be and they would notice and be concerned if this ever seemed different:

The texture of the medicine is another factor that may be used to determine whether or not it is real. When they are real, medications like paracetamol are difficult to break, but when they are fake or altered with, they are easily broken into smaller pieces. (<35-year-old male, rural Western Area Rural, Sierra Leone)

A few respondents in Ghana who were illiterate described an additional practice of asking literate members of the community to check the expiry dates of the medicines they were about to purchase or had just purchased before they consumed them:

Anytime I go to buy drugs I check the dates. I can’t read, so I mostly give it to someone to read and crosscheck the date to verify first. (39-year-old female, urban Upper East, Ghana)

Participants in Nigeria and Uganda also noted the added difficulty that illiteracy posed for following the campaign’s advice to only get medicines from licensed medicine sellers. Several participants noted that it was challenging to determine which medicine sellers were licensed, and illiteracy only added to this difficulty:

I have never gone to school before. How will I know that this is the correct license? (58-year-old female, rural Mbale, Uganda)

## Discussion

**Figure 3.**
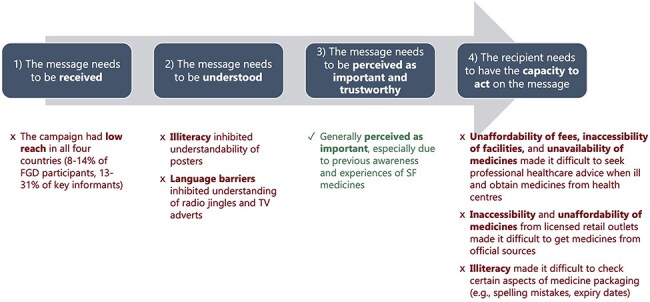
Summary of campaign evaluation findings mapped onto the analytical framework.

The aim of this study was to evaluate a pilot SF risk communication campaign, based on the framework that for a campaign to be effective it needs to fulfil four steps in relation to the target audience: (i) to reach them, (ii) be understood, (iii) be perceived as important, and (iv) be actionable (Fig. 1). Our evaluation suggests that only one of these four criteria was met (Fig. 3): participants generally perceived the materials and messages to be important and trustworthy, particularly in the contexts of their own personal experiences with suspected SF medical products. The campaign’s reach was low, and understandability was limited due to illiteracy and language barriers. A major limitation of the campaign was that participants faced substantial constraints on their capacity to act on the campaign’s advice due to unaffordability, unavailability, and inaccessibility of medicines from health facilities and licensed retail outlets, and illiteracy to check written information on medicines. These constraints are pervasive in sub-Saharan Africa and have been widely reported elsewhere (Okeke and Okeibunor 2010, Afari-Asiedu et al. 2018, Hamill et al. 2019, Tusubira et al. 2020).

Importantly, this evaluation has highlighted the potential for risk communication campaigns to widen or reinforce health inequities. Marginalized groups were generally excluded or disadvantaged in three of the four steps in the above framework. The campaign’s reach was limited overall, but recipients were predominantly men living in urban areas, while women living in rural areas were least likely to have encountered campaign materials. Broadly speaking, those who were less likely to understand the campaign due to illiteracy or language barriers were women, rural-dwellers, those with low education, older people, and those of minority ethnic groups who spoke minority languages. Finally, while many participants reported limitations on their capacity to act on the campaign’s messages, challenges were particularly salient for marginalized groups. For example, those living in rural areas faced difficulties with long distances to the nearest health facility or licensed medicine retailer, and those of lowest socioeconomic status found it especially challenging (and, at times, impossible) to afford consultation fees in the public sector and/or medicines in the private sector. Thus, health inequities are likely to be intensified by campaigns such as this unless equitability of reach, of understandability, and of capacity to act are intentionally prioritized and addressed.

To our knowledge, no literature has previously considered the equity implications of risk communication campaigns. A small number of studies have highlighted inequalities in health communications, especially during public health emergencies, showing that marginalized groups tend to be less likely to receive relevant messages. For example, systematic reviews of communication gaps during the H1N1 and COVID-19 pandemics showed that those with lower education and those of lower socioeconomic status were less likely to have received and/or understood public health communications and were more likely to experience poor outcomes (Lin et al. 2014, Häfliger et al. 2023). However, this literature has suggested that communication inequalities are the mechanism underpinning the link between social determinants of health and adverse health outcomes; this implies that addressing communication inequalities would largely resolve or avoid generating health inequities. We argue it is not that simple. Rather, we suggest that risk communication campaigns can widen health inequities by creating communication inequalities (i.e. campaigns tending to reach and be understood by more privileged groups) and by exacerbating structural inequities (i.e. campaign messages being more easily actionable by more privileged groups).

The latter point—that acting on the campaign’s messages was challenging, especially for marginalized groups—raises a key point regarding the likely utility of risk communication campaigns in this context. Campaigns can only be a solution to the extent that lack of awareness is the problem. Our previous work, based on representative samples surveyed in these same four countries, showed that the vast majority of respondents (>77%) were aware of SF medicines. Moreover, subgroups of the population (e.g. those with disability/illness in their household) who had greater awareness of SF medicines were also more likely to engage in ‘risky’ practices, such as buying medicines from unofficial sources (Wagnild et al. 2024). This suggests that, while there may be some gaps in awareness within the population, the more pertinent issue may be limitations on acting on information. Structural barriers such as unaffordability and unavailability of medicines impede access to quality medicines, and risk communications cannot address these. Unless and until these barriers are addressed through measures such as reduction of medicine stockouts in the public sector, removal or reduction of user fees in health facilities, and expansion of community health services in rural areas, campaigns simply cannot effect meaningful change. Furthermore, disseminating SF risk communications without simultaneously addressing structural issues runs the risk of victim-blaming, whereby individuals are empowered with information but unable to implement it and are blamed for what is ultimately a structural failure.

In addition to addressing structural barriers to medicine access, supply-side interventions to prevent SF medical products from reaching consumers are also required. Even if a risk communication campaign was effective (and equitable) in terms of reach, understandability, and capacity to act, the actions advised in the campaign can only reduce, not eliminate, the risk of encountering an SF product. For example, checking medicines and getting them only from official sources are important steps but they do not guarantee protection: SF medical products are not always visually detectable and can be present even in licensed outlets in both public and private sectors (Almuzaini et al. 2013). Thus, efforts to secure supply chains and tighten regulation are additionally required to protect consumers from SF products.

### Strengths and limitations

This study used a predominantly qualitative design to provide an in-depth evaluation of the pilot campaigns and to allow contextual understanding of the issues related to medicine access within each study site. Conducting fieldwork with diverse groups in urban and rural sites from contrasting regions within each country also ensured broad ranges of views and circumstances were captured. However, this study is not without limitations. The estimates of the campaign’s reach cannot be generalized as the sampling strategy was purposive. It is possible that the campaign’s reach was higher (or lower) in areas of each country that were not included in the evaluation. Caution should be exercised in generalizing the findings beyond these four (Anglophone) countries, although the fact that the findings were similar across the four countries suggests there is a broader pattern here which is likely to pertain across other similar (LMIC/African) contexts.

## Conclusion

This evaluation of pilot risk communication campaigns has key implications for public health campaigns as well as policies to address SF medical products. First, we have shown that the effectiveness of this campaign was severely limited by low reach, limited understandability, and lack of capacity for many to follow the campaign’s messages. Second, this campaign (like other public health awareness-raising campaigns) is likely to widen health inequities because disadvantaged groups (including rural-dwellers, women, those of lower socioeconomic status, and those with low education) were less likely to receive, understand, and be able to respond to the campaign’s messages. As these groups are already disproportionately impacted by SF medical products, the risk of widening inequities through risk communication campaigns is high. We recommend that public health campaigns carefully consider their likely impacts on equity before implementation and prioritize pro-equitability approaches wherever possible. Given that campaigns will only ever be effective if the public have the capacity to act on the campaign’s advice, interventions to improve access to quality medicine through health systems strengthening and increasing regulatory capacity should precede (or go hand-in-hand with) any subsequent awareness-raising efforts.

## Supplementary Material

czaf004_Supp

## Data Availability

The data underlying this article will be shared on reasonable request to the corresponding author.

## Reflexivity statement

As an international research project, drawing on data and insights from multiple countries, the authors on this paper are diverse in terms of regional location, seniority, gender, and race/ethnic identity. The first author is a junior scholar. Three authors, including the first and final authors, are women. Ten of the 13 authors are based in the Global South (Ghana, Nigeria, Uganda, Sierra Leone, and Republic of Congo). Eleven of the authors are people of colour. Our research team is committed to inclusive global scholarship and representation.
