# Supplementary material for: Can public education campaigns equitably counter the use of substandard and falsified medical products in African countries?
Source: Health Policy Plan. 2025 Jan 18;40(4):447–58. doi: 10.1093/heapol/czaf004 (PMC11979590; doi:10.1093/heapol/czaf004)
Supplement: czaf004_Supp [file czaf004_supp.zip › Supplementary File_revised.docx]

**
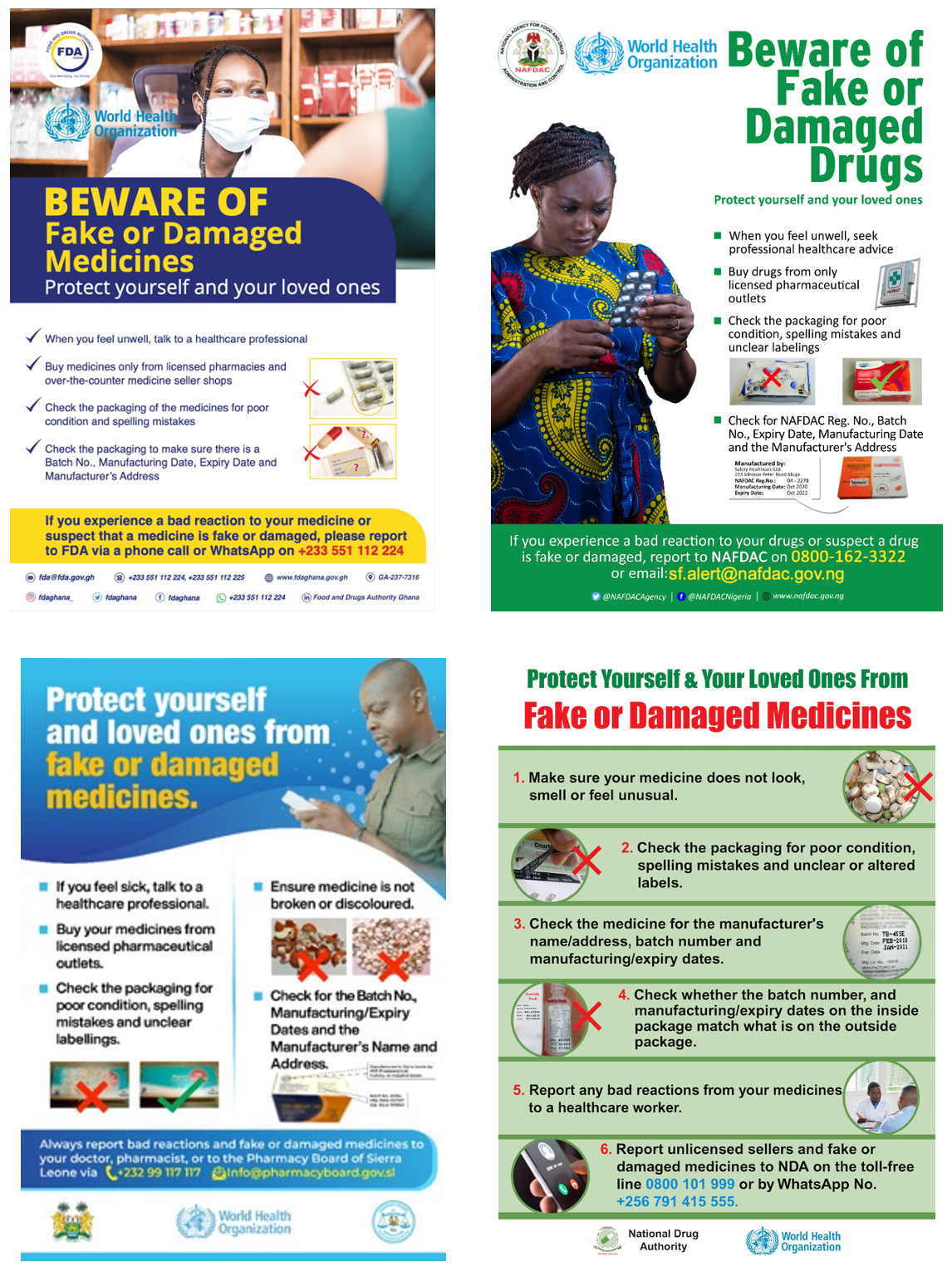
Figure S1.** Public-facing posters used in the campaign (clockwise from top left: Ghana, Nigeria, Uganda, Sierra Leone)

**Table S1.** Summary of the campaign contents and languages used in each of the four countries.

|  | **Ghana** | **Nigeria** | **Sierra Leone** | **Uganda** |
| --- | --- | --- | --- | --- |
| **Public-facing Poster** | ✓ English | ✓ English | ✓ English | ✓ English |
| **Healthcare Professional Poster** | ✓ English | ✓ English | ✓ English | ✓ English |
| **Radio Jingle/Talk** | ✓ Twi | ✓ English  ✓ Yoruba  ✓ Hausa  ✓ Igbo  ✓ Pidgin | ✓ English  ✓ Krio  ✓ Mende  ✓ Temne | ✓ Luganda  ✓ Lunyankore  ✓ Kiswahili  ✓ Lusoga  ✓ Lunyoro  ✓ Lugisu  ✓ Luo  ✓ Langi  ✓ Lugbara |
| **Television Advert** | ⅹ | ✓ English | ✓ English  ✓ Krio | ✓ English  ✓ Luganda |

**Table S2.** Fieldwork sites in the study.

| ***Ghana*** |  |  |
| --- | --- | --- |
| **Ecological zone** | **Administrative region (Municipality)** | **Study sites** |
| Savannah | Upper East (Bolgatanga) | Zuarungu (urban)  Bolgatanga (rural) |
| Coastal | Greater Accra (Ga South) | Ngleshie Amanfro (urban)  Kofi Kwei (rural) |
| ***Nigeria*** | | |
| **Geo-political zone** | **State/territory** | **Local government areas** |
| Central | Federal Capital Territory | Abuja Municipal Area Council (urban)  Bwari Area Council (rural) |
| North Central | Kwara | Ilorin West (urban)  Edu Local Government Area (rural) |
| ***Sierra Leone*** | | |
| **Region** | **District** | **Sub counties** |
| Western | Western Area Rural | Waterloo (urban)  Tombo (rural) |
| North | Port Loko | Port Loko (urban)  Mange Bureh (rural) |
| South | Bo | Bumpe (rural) |
| East | Kenema | Kenema (urban)* |
| ***Uganda*** | | |
| **Region** | **District** | **Study sites** |
| Western Ankole | Mbarara | Kakoba Division (urban)  Bubaare (rural) |
| Eastern Bugisu | Mbale | Northern Division (urban)  Bukasakya (rural) |

* No FGDs conducted here

**Table S3.** Focus group discussion guide.

1. **Project introduction (summarising the project, house-keeping, confidentiality) and seeking consent**
2. **Participant introductions and collection of background information** (e.g., gender, occupational status)
3. **Where do people get medicines and why there?**
   - Where do they usually get any medicines they or their household members may need? Why do they go where they go?
   - Do they ever experience difficulties getting the medicines they or their family need? If so, what kinds of difficulties?
   - How confident are they that the medicines they get are of good quality? How do they know?
4. **What do participants know about SF medical products and how?**
   - What have they heard from different sources?
   - Do they have any direct or indirect experience of potentially SF products?
   - Do they feel it’s something that affects them? Is it important to them? Is it a source of concern?
5. **Have participants seen any of the risk communications from the WHO-led project?** [Ask each individual to respond and tabulate responses]

***[Show the materials to all participants]***

1. **Discuss reactions to the materials and messages**
   - What are their reactions to the campaign materials?
   - What do they think the materials are trying to say?
2. **Discuss potential impacts of the messages on medicine-related behaviours**
   - If this message was shown more broadly, what do they think other people would do in response? Would other people understand it? Would it change anything about what people do?
   - What things might make it easier for people to respond to these messages? What things might make it harder?
3. **Discuss the possibility of (negative) unforeseen consequences of the campaigns**
   - Might people change their behaviour in a worse way rather than a better way?
   - Are there any other possible unforeseen consequences?
   - Overall, what do you think the impact of showing these materials might be?
4. **Ask about awareness of previous medicine incidents/recalls**

- Have people ever heard of cases/incidents where a medicine has been recalled?
- How did people hear about such incidents?
- How did they react to the news? Did it cause any changes in their thoughts or actions related to medicines?

1. **Brainstorm next steps**
   - Going forward, what do you think the most sensible thing to do is to help ensure that the medicines people get are good quality?
   - [If people mention communication campaigns as potentially useful or important], how should the messages be presented to be most meaningful and accessible?
2. **Closing discussion**
   - Follow up on any questions, threads and leads to get more depth as required
   - Ask for any additional comments, thoughts and reflections
   - Thank the participants and close

**Table S4.** Key informant interview guide/checklist of information to capture at each site.

**Where people get medicines and why they go where they go:**

- Where do people in this community get medicines?
- Which places are more commonly patronized than others and why? (Potentially touching on issues of convenience, cost, distance, stock-outs, trust)
- Are medicines sold in informal markets in this community? Do peddlers come to this community to sell medicines?
- How many pharmacies/OTC shops/health centres/etc are in the community and where are they?

**Whether the campaign materials have been seen by the informant and whether the informant thinks the community has seen/heard them:**

- Have you personally seen/heard these materials? If so, where/when?
- Do you think people in the community have seen/heard these materials?

**What impact the campaign materials might have and what things might make it easier or more difficult for people to act on the messages**

- What is/was your reaction to the messages in the campaign materials?
- Do you think it is realistic for the people in this community to follow the advice in the campaign? Why or why not?
- What might make it difficult for people to respond to the advice in the campaign?
- What might make it easier for people to respond to the advice in the campaign?
- Is public education like this useful/important? What (if any) are its limitations?
- In your view, what would make a campaign like this more effective going forward?

**Table S5.** Description of key informants.

|  | | | | | | | **Total** |
| --- | --- | --- | --- | --- | --- | --- | --- |
| **Ghana** | | | | | | | **21 (18.9%)** |
| **Greater Accra (n=10)** | | **Upper East (n=11)** | |  |  |  |  |
| *Urban (n=5)* | *Rural (n=5)* | *Urban (n=8)* | *Rural (n=3)* |  |  |  |  |
| Community leaders *(n=3)* | Community leaders *(n=2)* | Community leaders *(n=2)* | Community leader |  |  |  |  |
| Healthcare professional | Religious leader | Religious leaders *(n=2)* | Healthcare professional |  |  |  |  |
| Medicine retailer | Healthcare professional | Healthcare professionals *(n=2)* | Medicine seller |  |  |  |  |
|  | Medicine retailer | Medicine retailers *(n=2)* |  |  |  |  |  |
|  | | | | | | |  |
| **Nigeria** | | | | | | | **35 (31.5%)** |
| **Federal Capital Territory (n=18)** | | **Kwara State (n=17)** | |  |  |  |  |
| *Urban (n=10)* | *Rural (n=8)* | *Urban (n=9)* | *Rural (n=8)* |  |  |  |  |
| Healthcare professionals *(n=7)* | Healthcare professionals *(n=7)* | Healthcare professionals *(n=7)** | Healthcare professionals *(n=2)* |  |  |  |  |
| Community leaders *(n=3)* | Community leader | Community leaders *(n=2)* | Community leader |  |  |  |  |
|  |  |  | Medicine retailers *(n=5)†* |  |  |  |  |
|  | | | | | | |  |
| **Sierra Leone** | | | | | | | **39 (35.1%)** |
| **Western Area Rural District (n=7)** | | **Port Loko District (n=19)** | | **Bo District (n=12)** | | **Kenema District (n=1)** |  |
| *Urban (n=3)* | *Rural (n=4)* | *Urban (n=17)* | *Rural (n=2)* | *Urban (n=2)* | *Rural (n=10)* | *Rural (n=1)* |  |
| Community leader | Community leaders *(n=2)* | Community leader | Community leader | Community leader | Community leaders (n=8)† | Hospital pharmacist |  |
| Religious leader | Religious leader | Religious leaders *(n=2)* | Health professional | District pharmacist | Religious leader |  |  |
| Medicine retailer | Medicine retailer | Health professionals *(n=14)‡* |  |  | Health professional |  |  |
|  | | | | | | |  |
| **Uganda** | | | | | | | **16 (14.4%)** |
| **Mbale (n=8)** | | **Mbarara (n=8)** | |  |  |  |  |
| *Urban (n=4)* | *Rural (n=4)* | *Urban (n=4)* | *Rural (n=4)* |  |  |  |  |
| Community leaders (n=2) | Local leader | Medicine retailers (n=2) | Hospital in-charge |  |  |  |  |
| Midwife | Nurse | Pharmacist | Nurse/medicine seller |  |  |  |  |
| VHT | VHT | Nurse/medicine seller | Health worker |  |  |  |  |
|  | Health worker |  | Medicine retailer |  |  |  |  |

*Five of the seven were interviewed as a group; †Interviewed as a group ; ‡Thirteen of fourteen were interviewed as a group; VHT: Village Health Team worker

**Table S6**. Reach of the campaign materials amongst FGD participants.

|  | **Saw poster** | **Heard radio jingle/show** | **Saw TV advert** |
| --- | --- | --- | --- |
| **Ghana (n=137)** | **5 (3.6%)** | **6 (4.4%)** | **NA** |
| *Greater Accra* |  |  |  |
| Urban (n=29) | 4 (2.9%) | 5 (3.6%) | NA |
| Rural (n=27) | 1 (0.7%) | 0 (0.0%) | NA |
| *Upper East* |  |  |  |
| Urban (n=36) | 0 (0.0%) | 1 (0.7%) | NA |
| Rural (n=45) | 0 (0.0%) | 0 (0.0%) | NA |
|  |  |  |  |
| **Nigeria (n=174)** | **1 (0.6%)** | **13 (7.5%)** | **6 (3.4%)** |
| *Federal Capital Territory* |  |  |  |
| Urban (n=48) | 1 (0.6%) | 4 (2.3%) | 2 (1.1%) |
| Rural (n=64) | 0 (0.0%) | 5 (2.9%) | 3 (1.7%) |
| *Kwara State* |  |  |  |
| Urban (n=48) | 0 (0.0%) | 4 (2.3) | 1 (0.6%) |
| Rural (n=14) | 0 (0.0%) | 0 (0.0%) | 0 (0.0%) |
|  |  |  |  |
| **Sierra Leone (n=115)** | **8 (7.0%)** | **7 (6.1%)** | **1 (0.9%)** |
| *Western Area Rural District* |  |  |  |
| Urban (n=27) | 7 (6.1%) | 0 (0.0%) | 0 (0.0%) |
| Rural (n=21) | 0 (0.0%) | 0 (0.0%) | 1 (0.9%) |
| *Port Loko District* |  |  |  |
| Urban (n=16) | 1 (0.9%) | 3 (2.6%) | 0 (0.0%) |
| Rural (n=33) | 0 (0.0%) | 0 (0.0%) | 0 (0.0%) |
| *Bo District* |  |  |  |
| Rural (n=18) | 0 (0.0%) | 4 (3.5%) | 0 (0.0%) |
|  |  |  |  |
| **Uganda (n=185)** | **9 (4.9%)** | **0 (0.0%)** | **15 (8.1%)** |
| *Mbale* |  |  |  |
| Urban (n=51) | 3 (5.9%) | 0 (0%) | 5 (9.8%) |
| Rural (n=53) | 6 (11.3%) | 0 (0%) | 6 (11.3%) |
| *Mbarara* |  |  |  |
| Urban (n=39) | 0 (0%) | 0 (0%) | 2 (5.1%) |
| Rural (n=42) | 0 (0%) | 0 (0%) | 2 (4.8%) |
|  |  |  |  |
| **Total (n=611)** | **23 (3.8%)** | **26 (4.3%)** | **22 (4.6%)** |

Within-country data shown as n (%) of FGD participants within each country; the percentages shown in the total (bottom) row are proportions of all FGD participants (n=611), except for the TV advert which is shown out of 474 because there was no TV advert in Ghana.

**Table S7**. Reach of the campaign materials amongst key informants.

|  | **Saw poster** | **Heard radio jingle/show** | **Saw TV advert** |
| --- | --- | --- | --- |
| **Ghana (n=21)** | **3/21 (14.3%)** | **4/21 (19.0%)** | **NA** |
| *Greater Accra* |  |  |  |
| Urban (n=5) | 0/5 (0.0%) | 1/5 (4.8%) |  |
| Rural (n=5) | 1/5 (4.8%) | 1/5 (4.8%) |  |
| *Upper East* |  |  |  |
| Urban (n=8) | 2/8 (9.5%) | 2/8 (9.5%) |  |
| Rural (n=3) | 0/3 (0.0%) | 0/3 (0.0%) |  |
|  |  |  |  |
| **Nigeria (n=35)** | **1/35 (2.9%)** | **7/19 (36.8%)** | **8/22 (36.4%)** |
| *Federal Capital Territory* |  |  |  |
| Urban (n=10) | 0/10 (0.0%) | 3/7 (42.9%) | 4/7 (57.1%) |
| Rural (n=8) | 0/8 (0.0%) | 2/7 (28.6%) | 1/6 (16.7%) |
| *Kwara State* |  |  |  |
| Urban (n=9) | 1/9 (11.1%) | 1/3 (33.3%) | 1/2 (50.0%) |
| Rural (n=8) | 0/8 (0.0%) | 1/2 (50.0%) | 2/7 (28.6%) |
|  |  |  |  |
| **Sierra Leone (n=39)** | **3/39 (7.7%)** | **5/39 (12.8%)** | **2/39 (5.1%)** |
| *Western Area Rural District* |  |  |  |
| Urban (n=3) | 1/3 (2.6%) | 1/3 (2.6%) | 0/3 (0.0%) |
| Rural (n=4) | 0/4 (0.0%) | 0/4 (0.0%) | 0/4 (0.0%) |
| *Port Loko District* |  |  |  |
| Urban (n=17) | 0/17 (0.0%) | 1/17 (2.6%) | 0/17 (0.0%) |
| Rural (n=2) | 1/2 (2.6%) | 1/2 (2.6%) | 1/2 (2.6%) |
| *Bo District* |  |  |  |
| Urban (n=2) | 1/2 (2.6%) | 0/2 (0.0%) | 0/2 (0.0%) |
| Rural (n=10) | 0/10 (0.0%) | 2/10 (5.1%) | 1/10 (2.6%) |
| *Kenema District* |  |  |  |
| Rural (n=1) | NA | NA | NA |
|  |  |  |  |
| **Uganda (n=16)** | **0/16 (0%)** | **0/10 (0%)** | **2/10 (20%)** |
| *Mbale* |  |  |  |
| Urban (n=4) | 0/16 (0%) | NA | NA |
| Rural (n=4) | 0/16 (0%) | NA | NA |
| *Mbarara* |  |  |  |
| Urban (n=4) | 0/16 (0%) | 0/5 (0%) | 2/5 (40%) |
| Rural (n=4) | 0/16 (0%) | 0/5 (0%) | 0/5 (0%) |
|  |  |  |  |
| **Total (n=111)** | **7/111 (6.3%)** | **16/89 (18.0%)** | **12/71 (16.9%)** |

Within-country data shown as n (%) of key informants within each country; the percentages shown in the total (bottom) row are proportions of all key informants. All key informants were shown the poster but not all were shown the TV advert or radio talk show due to time or resource constraints; the denominator reflects the number shown the materials. NA indicates participants were not shown a particular campaign material.
